# Diabetes Distress and Health-Related Quality of Life among Patients with Type 2 Diabetes—Mediating Role of Experiential Avoidance and Moderating Role of Post-Traumatic Growth

**DOI:** 10.3390/ijerph21101275

**Published:** 2024-09-25

**Authors:** Chinenye Joseph Aliche, Erhabor Sunday Idemudia

**Affiliations:** Faculty of Humanities, North-West University, Potchefstroom 2520, South Africa; erhabor.idemudia@nwu.ac.za

**Keywords:** emotional distress, health-related quality of life, patients with type 2 diabetes, positive adaptation, avoidance behaviour

## Abstract

Objectives: Many people living with type 2 diabetes experience diabetes distress which impacts negatively on their health-related quality of life (HRQoL). However, little is known about the modifiable factors or psychological processes that make this happen. The current study examines the role of experiential avoidance and post-traumatic growth (PTG) in association with diabetes distress and the HRQoL. Methods: Participants included 303 patients with type 2 diabetes conveniently selected from two tertiary healthcare institutions in Nigeria. They completed relevant self-report measures. The Hayes PROCESS macro for SPSS was used for data analysis. Results: The results showed that experiential avoidance mediated the association between diabetes distress and the HRQoL [95% CI: −0.15, −0.07]. PTG significantly moderated the association between diabetes distress and the HRQoL [95% CI: 0.01, 0.02]. Specifically, diabetes distress was associated with a poor HRQoL only among patients with low levels of PTG but not among those with average and high levels of PTG. Conclusion: These findings underscore the importance of Acceptance and Commitment Therapy as it can potentially decrease the experiential avoidance behaviour of patients. Moreover, intervention should also target the facilitation of PTG due to its beneficial effects in reducing the negative effects of diabetes distress on health and recovery.

## 1. Introduction

There has been an overwhelming global increase in the prevalence of type 2 diabetes mellitus (T2DM) in recent times due to Western dietary habits, increased stress, reduced physical activity and prolongation of life expectancy [1]. In Nigeria, there are over 2.7 million cases of diabetes mellitus (DM) [2]. Out of this number, T2DM is the most common, as it constitutes over 90% of all diabetes cases, thereby making T2DM a major public health issue that requires urgent national attention in Nigeria [2]. People diagnosed with T2DM require a lifetime self-management plan and complex care activities such as a healthy diet, physical exercise, regular blood glucose monitoring and long-term medication [3]. Diabetes may impact their daily routine and increase their level of dependence and discomfort. This kind of regimented lifestyle due to diabetes may cause patients to experience negative emotions, including depression, anger, frustration and loneliness [4]. In addition, there may be concern about complications occurring, diabetes-related distress, experiential avoidance of self-care and death-related anxiety, which may impact their health-related quality of life (HRQoL) [2]. Beyond these psychosocial challenges, scientific investigations have shown that patients with T2DM may also experience positive adaptation to their illness, such as post-traumatic growth (PTG) [5,6,7]. Together, these psychosocial factors may directly or indirectly impact the HRQoL of patients with T2DM [2,3,4,8]. The HRQoL reflects the extent to which patients’ happiness and satisfaction with life are impacted by their health condition and treatment [8]. There is a need to optimise the HRQoL of patients with T2DM. To that end, the present study sought to examine the role of certain psychosocial factors, such as diabetes-related distress, experiential avoidance and posttraumatic growth, on the HRQoL among patients with T2DM.

Individuals diagnosed with T2DM experience a great deal of distress as they struggle to cope with the challenges associated with their illness. Evidence revealed that about a quarter of patients with T2DM in developed countries experienced diabetes distress [9], and this number is expected to be higher in developing countries [10]. Diabetes distress refers to a psychological condition reflecting the burden and stress associated with coping with diabetes [4], and this can occur in four different forms: physician-related, emotional, regimen-related and interpersonal-related distress, together representing multiple sources of diabetes distress [11]. Coping with the demands of diabetes and its treatment regimen can be very stressful, and previous studies have reported that diabetes distress is associated with a poor HRQoL among patients with T2DM [4,12]. However, the mechanism through which diabetes distress leads to a poor HRQoL is not clear. Thus, researchers and healthcare providers have acknowledged that the coping strategy utilised by patients with diabetes-related distress is very critical in determining their psychological health outcome [13]. While some patients may utilise adaptive coping strategies such as acceptance and positive refraining, others may engage in avoidance behaviours such as experiential avoidance [1,13]. Therefore, one of the goals of the current study is to examine whether experiential avoidance would potentially mediate the relationship between diabetes-related distress and the HRQoL among individuals living with T2DM.

Experiential avoidance reflects an individual’s unwillingness to remain in contact with unpleasant experiences, including body sensations, thoughts, emotions and memories associated with their health conditions, and may take action towards altering those unpleasant experiences or events that evoke them, including all forms of avoidance and escape [14]. Experiential avoidance is very common among patients with T2DM [2], and in modern cognitive behavioural interventions such as Acceptance and Commitment Therapy (ACT), experiential avoidance is perceived as a core psychopathological process. Specifically, ACT posits that an attempt at avoidance may help to decrease and alleviate emotional distress in the short term but could paradoxically reinforce and exacerbate the strength and frequency of unpleasant experiences and concomitant distress, especially when it is being used consistently over a long period of time [15]. So, ACT is an acceptance-based approach that helps patients to accept facts as they are instead of engaging in avoidance behaviour [16]. Thus, the main goal of ACT is to decrease experiential avoidance by promoting psychological flexibility, the tendency to perceive negative thoughts and emotions as an integral part of human existence with greater acceptance of those thoughts and less engagement in avoidance behaviour [16,17]. In other words, even though the diagnosis of diabetes and its treatment is a stressful life experience, the stress response to it varies according to patients’ cognitive evaluation of the health situation [13]. Higher experiential avoidance is associated with the maintenance of many psychological problems, including a poor HRQoL among people living with chronic disease [2,14].

Increased diabetes-related distress may fuel the tendency to engage in experiential avoidance as a way of coping with the health problem [1]. Previous studies have shown that experiential avoidance mediated the association between adverse psychological antecedence and health outcomes. For example, experiential avoidance mediated the relationship between diabetes distress and self-stigma in people with T2DM in the Republic of Korea [1]. Experiential avoidance mediated the association between emotion regulation abilities and loneliness [18], and the relationship between paranoid ideation and depressive symptoms [19], as well as the association between rumination and depression [20], illness appraisal and medication adherence [21], and the effects of maladaptive coping styles on psychopathology and mental health [22]. We hypothesize that experiential avoidance would mediate the association between diabetes distress and the HRQoL among patients with T2DM.

However, it is very critical to note that psychological changes associated with the experience of T2DM may not always be negative and pathological. There has been growing evidence revealing that people living with diabetes may also develop positive psychological adaptation to their illness, often referred to as post-traumatic growth (PTG) [5,7]. PTG reflects the positive psychological changes that occur as a result of the struggle to cope with a negative life experience, including diabetes [7]. Patients with T2DM who experience higher PTG have been able to articulate positive benefits and gains associated with their illness. These realisations and cognitive perceptions have been noted to contribute significantly to improving their health and well-being [5]. A higher PTG has been associated with an improved HRQoL among clinical samples [8,23].

Crucially, it is possible that a greater ability to experience PTG following a T2DM diagnosis may buffer the effect of diabetes distress on the HRQoL. Empirical studies have shown that a higher PTG is associated with a decreased experience of psychological distress [24,25] and that PTG is found to moderate the effects of negative psychosocial antecedence on better health outcomes among clinical and non-clinical samples. For example, PTG moderated the relationship between symptoms of posttraumatic stress disorder on adjustment and positive affective reactions among cancer patients [26]. PTG moderated the association between violent revictimisation and persisting PTSD symptoms in victims of interpersonal violence [27] and the effect of posttraumatic stress on the quality of life in U.S. military veterans with life-threatening illness or injury [28], and also moderated the relationship between secondary traumatic stress and burnout [29]. Based on the findings of these studies, it is worth noting that PTG is a very critical psychosocial factor that needs to be facilitated among individuals who have been exposed to stressful life events. In the present study, we do not know whether PTG would moderate the association between diabetes distress and the HRQoL among patients with T2DM. Generally, we hypothesised that the association between diabetes distress and the HRQoL of patients with T2DM would be [1] mediated by experiential avoidance and [2] moderated by PTG.

## 2. Materials and Methods

### 2.1. Participants

Participants in this study were 303 patients with T2DM conveniently selected from two tertiary healthcare institutions in Nigeria: the University of Nigerian Teaching Hospital, Ituku-ozala, Enugu state, and the Nnamdi Azikiwe University Teaching Hospital, Okofia, Anambra state, Nigeria. The eligibility criteria for participation include (1) adult > 18 years; (2) must have been diagnosed with T2 diabetes (which was confirmed through the patients’ personal file in the hospitals) and on medication; (3) must be mentally stable enough to be able to respond to the psychological instrument; (4) must be able to read and understand English language at least at the secondary school level; (5) must not be taken any antipsychotic medication as at the period of this study.

### 2.2. Instruments

#### 2.2.1. Diabetes-Related Distress

Diabetes-related distress was measured using the Diabetes Distress Scale (DDS) [30], a 17-item instrument that assesses diabetes-related distress among those living with diabetes. The inventory has four domains: emotional burden (e.g., “feeling overwhelmed by the demand of living with diabetes”), diabetes-related interpersonal distress (e.g., “feeling that my friends/family don’t appreciate how difficult living with diabetes can be”), physician-related distress (e.g., “feeling that my doctor doesn’t take my concerns seriously enough”) and regimen-related distress (e.g., “feeling that I am not sticking closely enough to a good meal plan”). Items of the inventory are rated on 6-point Likert scale ranging from 1 “not a problem” to 6 “a very serious problem”. The scores on the scale range from 17 to 102, with a higher mean score suggesting higher diabetes-related distress. The developers reported an internal consistency reliability coefficient (Cronbach’s alpha) of 0.93 for the total score, and a Cronbach’s alpha range of 0.83 to 0.85 was reported for the subscales [30]. In the current study, a total alpha reliability coefficient of 0.82 was obtained.

#### 2.2.2. Experiential Avoidance

Experiential avoidance was measured with the Acceptance and Action Questionnaire 11 (AAQ-11) [31]. This inventory is a 7-item self-report measure that assesses the tendency to avoid aversive internal experiences such as memories, thoughts, and negative emotions (e.g., my painful memories prevent me from having a fulfilling life). The items are scored on a 7-point Likert scale ranging from 1 = (never true) to 7 (always true), with scores ranging from 7 to 49, and a higher score indicates a greater EA. The developers reported an excellent construct validity and reliability coefficient value of 0.84, test-retest (r = 0.08 in 3 months) and r = 0.79 in 12 months [31]. The present study obtained an internal consistency reliability coefficient (Cronbach’s alpha) of 0.83.

#### 2.2.3. Posttraumatic Growth

Posttraumatic Growth was measured using the original 21-item post-traumatic growth inventory (PTGI) [32] that assesses the positive psychological changes that may occur following stressful life events. The PTGI has 5 subscales: relating to others, appreciation of life, new possibilities, personal growth and spiritual change. The items (e.g., “I am able to accept the way things work out” and “I have a greater appreciation for the value of my own life”) are rated on a 6-point Likert-type scale ranging from 0 = not at all, to 5 = very high degree, with scores ranging from 0 to 105, and a higher score suggesting higher levels of PTG. The PTGI has excellent psychometric properties [32] and has also demonstrated the same among Nigerian samples [33]. *In the present study, a Cronbach’s* alpha coefficient value of 0.83 was obtained for the global score.

#### 2.2.4. Health-Related Quality of Life

The HRQoL was assessed using the short form of the Diabetes Quality of Life Questionnaire (DQOLQ), which has 15 items derived from the original 60-item DQOLQ [34]. The participant’s responses on the items (e.g., “How satisfied are you with the amount of time it takes you to manage your diabetes”) are scored on a 5-point Likert scale ranging from 1 (very satisfied) to 5 (very dissatisfied), with lower scores suggesting improved diabetes-related quality of life. The DQOLQ has excellent validity with an internal consistency reliability (Cronback’s alpha) coefficient of 0.85, and test-retest reliability was found to be 0.513. The inventory has demonstrated a good convergent validity, being strongly and positively associated with the original 60-item DQOLQ full scale (r = 0.91), alongside the individual DQOLQ subscales: social worry subscale (r = 0.52) and satisfaction with diabetes control subscale (r = 0.97). The current study obtained an alpha coefficient value of 0.81.

### 2.3. Procedure

Following the approval of this study by the Research Ethics Committee of the University of Nigerian Teaching Hospital, Ituku-ozalla, Enugu State, Nigeria (Ethics Clearance Number: NHREC/75/02/08/2023), and the Nnamdi Azikiwe University Teaching Hospital Okofia, Anambra state, Nigeria (Ethics Clearance Number: NHREC/90/23/07/2023), data collection commenced with the help of 4 research assistants (2 in each hospital). They approached patients with diabetes who attended the hospital’s clinic between the months of August 2023 and March 2024. The patients with diabetes were educated on the main objectives of the study, including the confidentiality of their responses. Those who were available, willing and verbally consented to participate in the study were recruited. Those who met the inclusion criteria were given the psychological instrument, which they completed and returned immediately. Out of 320 questionnaires distributed, 303 were properly completed and returned and were used for data analysis.

### 2.4. Statistical Analysis

We carried out a preliminary analysis using descriptive statistics. Pearson’s correlation was employed in order to examine the relationship between the demographic variables (e.g., age, gender and educational status) and the main study variables. The Hayes PROCESS macro for SPSS was used to test the moderation and mediation hypothesis. Model 4 was used to test the mediation hypothesis, while Model 1 was used for moderation analysis. The 95% bootstrap confidence intervals CI were calculated using 5000 bootstrapped samples, and significant results were affirmed if the 95% CI did not include zero between the straddles [35]. All the data analysis was carried out using the Statistical Packages for Social Sciences (SPSS), version 23.

## 3. Results

The results in Table 1 showed that the participant’s ages ranged from 28 to 55: Mean = 38.09; SD = 6.57. Regarding their gender, 141 (48.5%) were male and 162 (53.5%) were female. For marital status, the majority of the participants, 221 (72.9%), were married and had a tertiary education, 180 (59.4%). Regarding their occupation, the greater proportion, 151 (49.9%), were predominantly farmers who rarely engage in regular exercise, 144 (47.5%). For the family history of diabetes, 161 (53.1%) had a family history, while 142 (46.9%) had no family history of diabetes. The results for the mean, standard deviation and correlations among the study variables are presented in Table 2 below.

### 3.1. Mediation Results

For the mediation analysis, the results showed that DD was positively associated with EA, {*B* = 0.37, t(301) = 6.43, *p* < 0.001}, and the overall model was significant, F (1, 301) = 41.31, *p* < 0.001, R^2^ = 0.12. We also found a significant negative effect of DD on the HRQoL {*B* = −1.10, t(301) = −2.85, *p* < 0.01}, and the overall model was significant, F (1, 301) = 8.11, *p* < 1.01, R^2^ = 0.03). When EA was introduced to the model, the previously significant association between DD and the HRQoL was no longer significant, {B = −0.03, t(300) = −1.96, *p* > 0.05). There was a significant negative relationship between EA and the HRQoL {*B* = −1.17, t(300) = −5.27, *p* < 0.001}, and the overall model was significant, F (2, 300) = 18.33, *p* < 0.001, R^2^ = 0.11. The test of indirect effect supported significant mediation (completely standardised test of indirect effect = −0.11, 95% CI [−0.15 to −0.07]) as the confidence interval did not include zero. The results indicate that EA fully mediated the relationship between DD and the HRQoL among patients with type 2 diabetes (see Table 3).

### 3.2. Moderation Results

**Figure 1:**
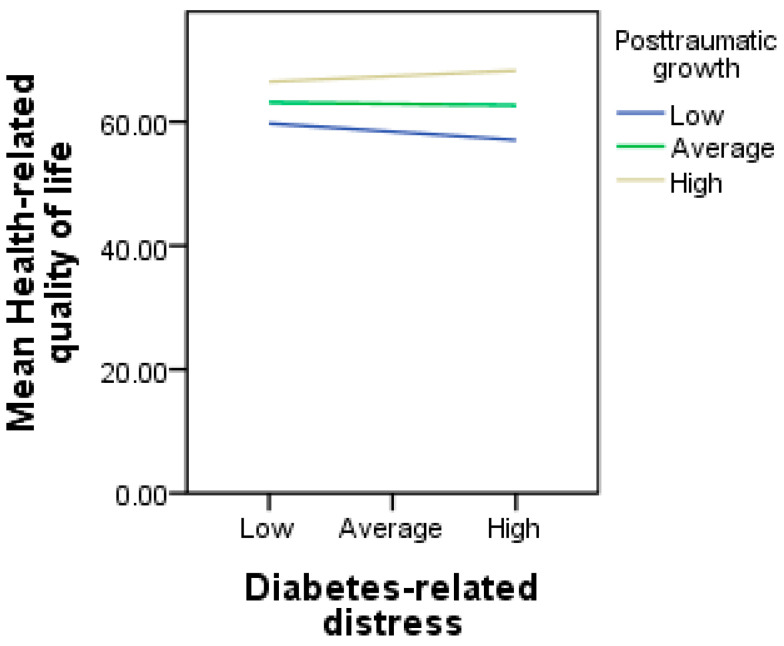
Showing the moderating role of posttraumatic growth in the relationship between diabetes-related distress and health-related quality of life.

Results on the moderating role of PTG in the relationship between DD and the HRQoL are presented in Table 4. The results showed that DD was not significantly associated with the HRQoL (B = −0.02, *p* = 0.616). PTG was positively associated with the HRQoL (B = 0.26, *p* = 0.000) and moderated the association between DD and the HRQoL (B = 0.02, *p* = 0.034). The overall model was significant, R = 0.49, R^2^ = 0.24, F (3, 299) = 21.38, *p* < 0.001. An examination of the interaction slope (Figure 1) revealed that DD was associated with a poor HRQoL only at low (B = −0.08, *p* = 0.040) but not at average (B = −0.01, *p* = 0.616) or high (B = 0.05, *p* = 0.220) levels of PTG. This indicates that PTG moderated the association between DD and the HRQoL, implying that a higher experience of PTG buffers the negative impact of DD on the HRQoL among patients with T2DM.

## 4. Discussion

The current study examined the mediating role of experiential avoidance in the relationship between diabetes distress and the HRQoL. It further explored whether PTG would moderate the association between diabetes distress and the HRQoL among individuals living with type 2 DM. The results revealed that experiential avoidance played a mediating role in the association between diabetes distress and the HRQoL. The impact of diabetes distress on the HRQoL of patients with T2DM is understood through a direct and indirect path (through experiential avoidance). The current research found that diabetes distress was negatively associated with the HRQoL. This result is in agreement with the findings of an earlier study, which showed that higher diabetes distress is related to a poor HRQoL [4]. An improved HRQoL entails that patients have a good general health perception, social relationship, cognitive functioning, greater energy, adequate sleep and adaptive coping competence [18]. Experiencing increased diabetes distress may negatively impact the patient’s perception of the effect of treatment and disease, which could, in turn, lead to a deterioration of the patient’s overall health and quality of life. Therefore, the negative effect of diabetes distress should be put into consideration even before developing interventions to promote an HRQoL of patients with diabetes.

The results also showed that experiential avoidance mediated the association between diabetes distress and the HRQoL. This suggests that the negative impact of diabetes distress on the HRQoL of patients with diabetes is transmitted through the mechanism of increased experiential avoidance. In other words, the extent to which diabetes distress is related to a poor HRQoL is a function of the individuals’ different tendency to unwillingly remain in contact with unpleasant experiences associated with the burden of diabetes. This is consistent with findings of previous related research showing that experiential avoidance partially mediated the link between diabetes distress and self-stigma among patients with type 2 diabetes in the Republic of Korea [1]. The psychological stress associated with coping with diabetes and the entire diabetes management protocols remains an unpleasant experience for patients with diabetes. In most cases, patients may unconsciously internalise these unpleasant experiences and feel emotional pains that inevitably result in heightened diabetes distress [4]. This kind of situation may lead to patients with diabetes engaging in experiential avoidance of self-care and other potential positive health behaviours that are very critical to facilitating effective treatment and recovery [21]. There is a high tendency that such patients with diabetes may also engage in rigid behavioural responses, including avoidance, self-doubt and denial as a way of coping with their illness. Unfortunately, spending an inordinate amount of time avoiding negative emotions and thoughts hinders the opportunity to pursue long-term goals and values, which are important indicators of an improved HRQoL [2,12].

Further to that, since controlling thoughts and suppressing unwanted emotions can be cognitively draining, higher experiential avoidance may potentially leave patients psychologically strained, leading to engagement in self-stigma [1]. This is because patients with diabetes with high experiential avoidance are less likely to appraise their health situations in a positive light and may have a low sense of meaning in life, which ultimately leads to a poor HRQoL [2]. Although the use of an experiential avoidance strategy may be helpful in coping with the discomfort of diabetes in the short term, higher and more frequent use of experiential avoidance, as triggered by increased diabetes distress, may lead to poor health outcomes and should never be encouraged [1]. Generally, the findings of this study highlight the underlying mechanism of the association between diabetes distress and the HRQoL by revealing that experiential avoidance may be an important target for psychological intervention strategies to improve the HRQoL of patients with diabetes.

Another noteworthy finding of this research is that PTG moderated the association between diabetes distress and the HRQoL. Specifically, higher diabetes distress was associated with a poor HRQoL among patients with diabetes with low levels of PTG but not those with average or high levels of PTG. This suggests that a greater experience of PTG was very protective as it buffered the negative impacts of diabetes distress on HRQoL of patients with T2DM. The significant interaction among these variables represents a complex relationship that previous studies have not been able to explore. However, studies have revealed that PTG moderated the effect of negative psychosocial factors on health outcomes [28,29]. Notably, many patients with diabetes often experience discomfort when they become fully aware of their disease and begin their medical treatment. This realisation can bring about a heightened awareness of the seriousness of the illness, resulting in serious concerns about how to survive [4]. These study findings have revealed that patients who are able to make positive meaning out of their struggle for survival by way of PTG experienced an improved HRQoL. One possible explanation for this result is that the PTG experience involves a transformational process that enables an individual to achieve greater awareness and functionality than before the diagnosis of a chronic illness. This kind of transformation is holistic, impacting positively on many areas of patients’ lives such that patients are more able to relate with other people very well, including other patients with diabetes who are coping very well with the illness and also healthcare providers by asking questions on what role they can play to improve their recovery [5,24]. Patients may also begin to appreciate their lives more, identify new possibilities in their lives, and experience increased personal strength and spiritual change [7]. These kinds of experiences enable patients to have a positive perception of diabetes management and a better understanding of the role they need to play to reduce distress and improve recovery and the HRQoL [8]. Therefore, the result on the moderating effect of PTG is a very novel contribution to the literature on diabetes care and management. It has provided evidence and clarification on how to cope with the negative effect of diabetes distress on the HRQoL of patients with diabetes.

### 4.1. Implications for Research and Clinical Practice

In general, the present study provides important theoretical and practical implications for the research field of the HRQoL of patients with diabetes. From a theoretical standpoint, this research not only initially confirmed the existence of diabetes distress linked to the HRQoL but also further explored the role of experiential avoidance and PTG as important mechanisms that influence the diabetes distress–HRQoL relationship. From a practical standpoint, this study offers healthcare professionals new insight to improve the significance of diabetes care and management by effectively reducing the tendency to engage in experiential avoidance and holistically promoting the experience of PTG among patients with T2DM. For example, Acceptance and Commitment Therapy (ACT) is a viable psychological intervention that may be useful in decreasing experiential avoidance [15]. ACT is an innovative behavioural treatment that incorporates mindfulness practices and acceptance-based intervention into its treatment package [15]. This therapeutic approach recognised that experiential avoidance, or escape from unwanted internal experiences, propels psychopathology and that dealing with experiential avoidance involves the facilitation of patients’ acceptance of themselves and their life experiences [16,17]. So, ACT may be employed to reduce the rigidity of thinking that characterised experiential avoidance and then facilitate diabetic patients’ level of psychological flexibility and acceptance attitude. With this, patients are well-positioned to relate comfortably to unpleasant thoughts and emotions, accept them as part of human lives and continue to pursue their life goals [17]. Furthermore, based on the finding that PTG buffered the effect of diabetes distress on the HRQoL of patients with diabetes, it has now become very imperative that healthcare professionals, including medical doctors, clinical psychologists and nurses, adopt an integrative approach to the care and management of diabetes. The target should be to determine how best to reduce the negative effect of diabetes distress and facilitate the development of PTG among patients with T2DM. Psychosocial therapies, such as mindfulness-based intervention, expressive-based and positive psychology-based approaches, can be very useful in that regard [36]. The facilitation of PTG using these interventions would not only allow patients with diabetes to overcome their trauma and distress but also result in them going over and above their pre-diabetes state, enhancing resilience, health and wellbeing [36]. Such interventions may begin with the assessment of patients’ levels of diabetes distress and perception of growth using the Diabetes Distress Scale (DDS) and the *Post-traumatic Growth Inventory (PTGI). Those with high scores in diabetes distress and those with low score in PTG might be the prime target for the psychological intervention.*

### 4.2. Limitations and Directions for Future Research

There are certain limitations of this study that need to be acknowledged. First, the current study utilised a cross-sectional design, which only allows for a cautious interpretation of its findings and precludes inferences regarding the direction of the relationship among the study variables. An experimental or longitudinal design could enhance the causal evaluation of similar phenomena in the future. A longitudinal study, in particular, could further help to track changes in the HRQoL of patients over time and the role that diabetes distress, experiential avoidance and PTG play in determining the outcome. Second, this study exclusively adopted self-report measures for data collection. Future studies may consider using multiple data sources such as behavioural measures, observer reports or data from experimental manipulation. Third, the researchers could not obtain data on certain clinical characteristics of the patients, such as duration of diabetes and common comorbidities, among others. Also, this study utilised a convenient sampling method, and data was collated among a single ethnic group in Nigeria (the Southeast Nigeria that constitutes the Igbo ethnic group). Thus, the sampling technique and lack of data on some clinical characteristics of patients, alongside the non-diversity of the samples, together may have directly or indirectly influenced the findings of this study. Future studies among diabetes patients should address these issues. There is also the need for future studies to explore the role of other psychosocial factors (e.g., mindfulness, positive mental health, meaning in life and intolerance of uncertainty) as possible mediators and moderators of the diabetes distress–HRQoL relationship. It would also be useful to examine the effectiveness of interventions targeting experiential avoidance and PTG in future studies.

## 5. Conclusions

The current study has contributed to the existing literature on the psychosocial factors related to HRQoL of patients with type 2 diabetes and established an empirical framework for researchers to examine the mediating role of experiential avoidance and the moderating role of PTG in the association between diabetes distress and the HRQoL. The results indicated that experiential avoidance is a key pathway in explaining why individuals with high diabetes distress experience a poor HRQoL. Moreover, results also showed that PTG moderated the relationship between diabetes distress and the HRQoL, such that higher diabetes distress was associated with poor HRQoL among patients with T2DM with low levels of PTG but not those with average and high levels of PTG. Therefore, interventions to promote the HRQoL of patients with diabetes should ultimately target experiential avoidance, and ACT is one effective psychological therapy that has shown some promise in decreasing the experiential avoidance behaviour of patients with diabetes. There is also the need to facilitate the development of PTG as it is capable of decreasing the impact of diabetes distress and ultimately promoting recovery and the HRQoL of patients with T2DM.

## Figures and Tables

**Table 1:** Demographic characteristics of participants (N = 303).

Age	Range 28–55	38.09 (6.57)
Gender	Male	141 (48.5)
	Female	162 (53.5)
Marital status	Single	34 (11.2)
	Married	221 (72.9)
	Widow(er)	48 (15.8)
Education	Secondary	123 (40.6)
	Tertiary	180 (59.4)
Occupation	Farmer	151 (49.9)
	Business	100 (33)
	Civil servants	17 (5.6)
	Students	13 (4.3)
	Others	22 (7.3)
Degree of exercise performed	Not perform any exercise	81 (26.7)
	Rarely	144 (47.5)
	Very often	78 (25.7)
Family history of diabetes	Has family history	161 (53.1)
	No family history	142 (46.9)

**Table 2:** Mean, Standard deviation and Correlations among the variables.

	Variables	1	2	3	4	5	6	Mean	SD
1.	Age	-						38.09	6.57
2.	Gender	0.06	-					-	-
3.	Education	0.06	0.08	-				-	-
4.	DD	0.05	0.04	−0.09	-			36.90	16.50
5.	EA	−0.15	0.08	0.01	0.35 ***	-		34.00	17.70
6.	PTG	0.04	−0.01	0.08	−0.23 ***	−0.45 ***	-	80.62	17.56
7.	HRQoL	0.04	−0.04	0.09	−0.16 ***	−0.33 ***	0.47 ***	52.63	9.99

Note: *** *p* < 0.001; Gender coded as 0 = male, 1 = female; Education coded as 0 = secondary, 1 = tertiary; DD = Diabetes-related distress; EA = Experiential avoidance; PTG = Posttraumatic growth, HRQoL = Health-related quality of life.

**Table 3:** Hayes PROCESS results of experiential avoidance mediating the relationship between diabetes-related distress and the HRQoL.

Model Paths	Beta	SE	*p*-Value	95% CI
Total effect	−0.10	0.03	0.004	[−0.17, −0.03]
Direct effect	−0.03	0.04	0.340	[−0.10, 0.04]
DD and EA	0.37	0.06	0.000	[0.26, 0.49]
EA and HRQoL	−0.17	0.03	0.000	[−0.24, −0.11]

**Table 4:** Hayes PROCESS results of post-traumatic growth moderating the relationship between diabetes-related distress and the HRQoL.

Variables	B	SE	T	*p*	95% CI
Diabetes-related distress (DD)	−0.02	0.03	−0.50	0.616	[−0.07, 0.04]
Post-traumatic growth (PTG)	0.26	0.04	7.09	0.000	[0.19, 0.33]
DD × PTG	0.02	0.01	2.13	0.034	[0.01, 0.02]

## Data Availability

The datasets generated and analysed during the current study are available from the corresponding author upon reasonable request.
